# Structural Anomaly in Glasses: Molecular Dynamics Study of Organic Radical in Dibutylphthalate at Different Temperatures

**DOI:** 10.3390/ijms232314859

**Published:** 2022-11-28

**Authors:** Dmitry V. Alimov, Mikhail Yu. Ivanov, Svetlana Pylaeva, Matvey V. Fedin

**Affiliations:** 1 International Tomography Center, Siberian Branch of the Russian Academy of Sciences, Novosibirsk 630090, Russia; 2 Physics Department, Novosibirsk State University, Novosibirsk 630090, Russia; 3 NMR Spectroscopy Group, Bijvoet Center for Biomolecular Research, Utrecht University, Padualaan 8, 3584 CH Utrecht, The Netherlands

**Keywords:** glasses, molecular dynamics, EPR

## Abstract

Understanding the heterogeneous nano/microscopic structures of various organic glasses is fundamental and necessary for many applications. Recently, unusual structural phenomena have been observed experimentally in various organic glasses near their glass transition temperatures ($T_{g}$), including dibutyl phthalate (DBP). In particular, the librational motion of radical probe in the glass is progressively suppressed upon temperature increase. In this work, we report in-depth molecular dynamics studies of structural anomalies in DBP glass, that revealed insights into the general mechanism of these phenomena. In particular, we have evidenced that the two types of solvation within alkyl chains coexist, allowing only small-angle wobbling of the solute molecule (TEMPO radical), and another favouring large-angle rotations. The former solvation assumes constrained location of the solute near carboxyl groups of DBP, while the latter is coupled to the concerted movement of butyl chains. Remarkably, excellent qualitative and quantitative agreement with previous experimental results were obtained. As such, we are certain that the above-mentioned dynamic phenomena explain the intriguing structural anomalies observed in DBP and some other glasses in the vicinity of $T_{g}$.

## 1. Introduction

It is well known that the majority of glasses consists of immobile “solid-like” and mobile “liquid-like” domains close to the glass transition temperature ($T_{g}$), and that these domains have significantly different dynamic properties [1,2,3,4]. To date, glass heterogeneity has been evidenced by plenty of techniques: X-ray and neutron scattering, optical spectroscopy, NMR and others [5,6,7,8,9]. In most cases, organic solvents able to form glasses upon cooling have been investigated.

Recently, unusual molecular mobility in ionic liquids (IL) near $T_{g}$ was observed and assigned to the structural rearrangements occurring on the nanometer scale in the glassy state. In particular, the coexistence of two types of IL environments was observed, one of which progressively suppresses the molecular mobility upon temperature increase between ≈($T_{g}-60$ K) and $T_{g}$. This is a highly uncommon behavior, since most known substances, including the same ILs in bulk, become less dense upon temperature increase, this was termed a “structural anomaly” [10]. Later on, it was demonstrated that such structural anomalies are governed by the alkyl chains of glass-forming compounds [11,12,13]. Furthermore, it was found that some non-IL compounds, such as those mimicking imidazolium cations, also exhibit similar structural anomalies. In particular, this was clearly evidenced experimentally for phthalates [14]. However, despite the clear role of alkyl chains, the mechanism of such anomalies is still far from being understood.

Molecular Dynamics (MD) simulations are and have been actively employed to understand the local structures and dynamics of various molecular liquids and their glasses [15,16,17,18,19,20,21,22,23,24,25,26,27]. Therefore, we used MD in an attempt to explain the structural anomalies previously observed in ILs [11] and phthalates [14]. The MD studies of ILs are more challenging compared to phthalates, because of the precise treatment required due to the charged nature of ILs. At the same time, since similar structural anomalies are also observed in neutral phthalates in a the glassy state, application of MD to such glasses might be both easier and more informative. Therefore, in this work we performed the first in-depth study of structural anomalies in dibutyl phthalate (DBP) and compared our results with previously obtained experimental data. The experiments were conducted using electron paramagnetic resonance (EPR) spectroscopy, where the mobility of DBP was measured vs. temperature [11,14]. Two types of motion—large-scale diffusive rotation and small-angle wobbling (below referred to as stochastic molecular librations) were identified and investigated. It was shown that at temperatures slightly below the glass transition, there was a density anomaly: formation of areas where the radical was more compressed and exhibits decreasing mobility with temperature (the anomaly), or areas where the radical is less compressed and its mobility grows with temperature (similar to many common glasses).

In this work, we carried out MD simulations, where the above suppression of librations at a temperature close to $T_{g}$ (DBP) ≈ 180 K [28,29] was studied theoretically. We examined the peculiarities of the mutual radical-solvent arrangement and the dependence of the radical mobility on its environment at different temperatures. The questions, addressed in this study were the following: (i) What molecular interactions are responsible for the structural anomaly? (ii) What interactions allow for different rotational freedom of the radical? (iii) Do similar motion patterns occur in bulk DBP?

## 2. Results and Discussion

**Figure 1:**
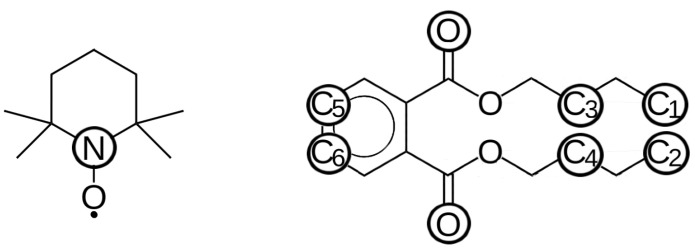
Structure of TEMPO and dibutyl phthalate molecules with positions and labels of atoms used in analysis.

**Figure 2:**
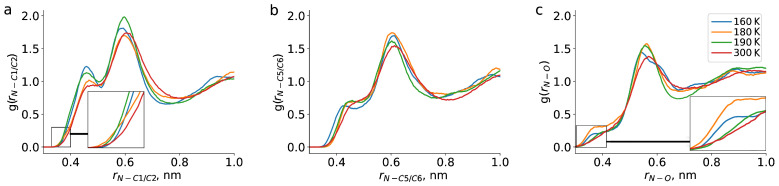
Radial distribution functions between carbon atoms of chain ends (C1 and C2) (**a**), rings (C5 and C6) (**b**), oxygen atoms (O) (**c**) of DBP and nitrogen atom of TEMPO (N) at different temperatures.

We probed the solvation of the TEMPO radical by various methods: radial distribution functions (RDFs, g(*r*)), rotational auto-correlation functions (RACF), etc. Atoms with corresponding labels that were used in the analysis are shown in Figure 1. RDFs from the nitrogen atom of TEMPO radical (N) to carbon atoms of the ring (C5 and C6), carbon atoms of the methyl group (C1 and C2) as well as oxygen atoms of carbonyl group (O) of the dibutyl phthalate (DBP) molecule are shown in Figure 2. The nitrogen atom of TEMPO was chosen as it is located very close to its center of mass. Based on the RDFs we conclude that TEMPO is predominantly solvated by alkyl chains of DBP.

The majority of the averaged properties of the solution did not show irregular behaviour with temperature. However, at short distances (shorter than ∼0.4 nm) important characteristics were found for the RDF from N atom of TEMPO to methyl carbons of DBP, g($r_{N-C1/2}$). In this case, the order of the curves for T = 160–300 K changed, and corresponding probability for such short-range structures became higher at 180 K than at other temperatures (Figure 2a). The same was true at short distances for the RDFs from N atom of TEMPO to carbonyl oxygens of the DBP g($r_{N-O}$). These curves also exhibited irregular behaviour: the peak at 0.37 nm has maximum intensity for 180 K, followed by 160 K (Figure 2c). For the 190 and 300 K the peak was shifted to larger distances (0.4 nm) and its intensity was smaller. These two findings agreed with previously reported changes in local density with temperature, i.e., the number of atoms of DBP in direct contact with TEMPO showed irregular behaviour and had a maximum value for 180 K trajectories [14]. Speaking of the RDFs calculated for the distances between N atom of radical and the DBP ring g($r_{N-C5/6}$) (Figure 2b), they did not display any noticeable irregularity in behaviour with temperature. This also agreed well with preferred orientation of alkyl-chain side of DBP toward the radical.

**Figure 3:**
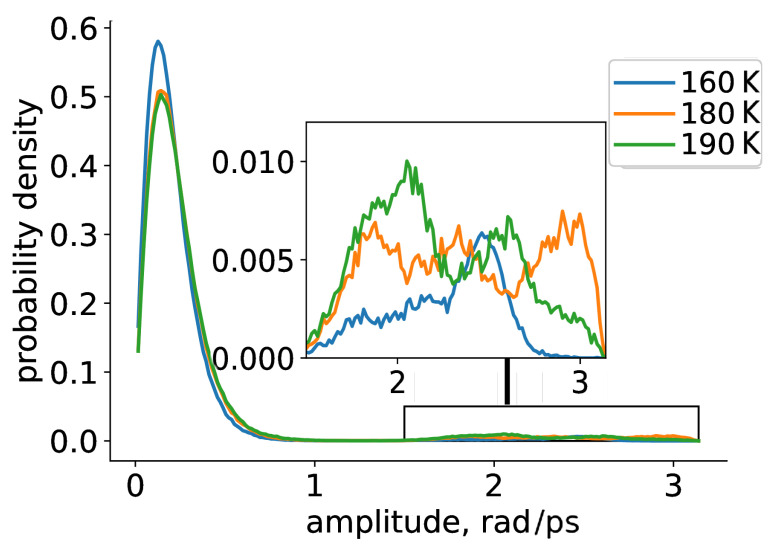
Rotational spectra of TEMPO radical at different temperatures.

Rotational spectra (Fourier transform of the rotational auto-correlation function) of the TEMPO radical at low temperature calculations are presented in Figure 3. The positions of peaks in such spectra and their intensities provided complementary information, reflecting the fraction of radicals exhibiting a particular rotational behaviour. For all three temperatures we observed that the majority of TEMPO radicals undergo small-angle librations (peak at ∼0.1 rad); however, some weak manifestations of large-scale rotations were also visible (inset to Figure 3). Remarkably, the intensity of the small-angle peak was smaller at 180–190 K relative to 160 K, whereas the intensity of large-scale rotation was larger. This is in excellent agreement with EPR results, which show the growth of mobile fraction (exhibiting diffusive rotation) at 160–190 K at the expense of decreasing immobile (librating) fraction [14].

**Figure 4:**
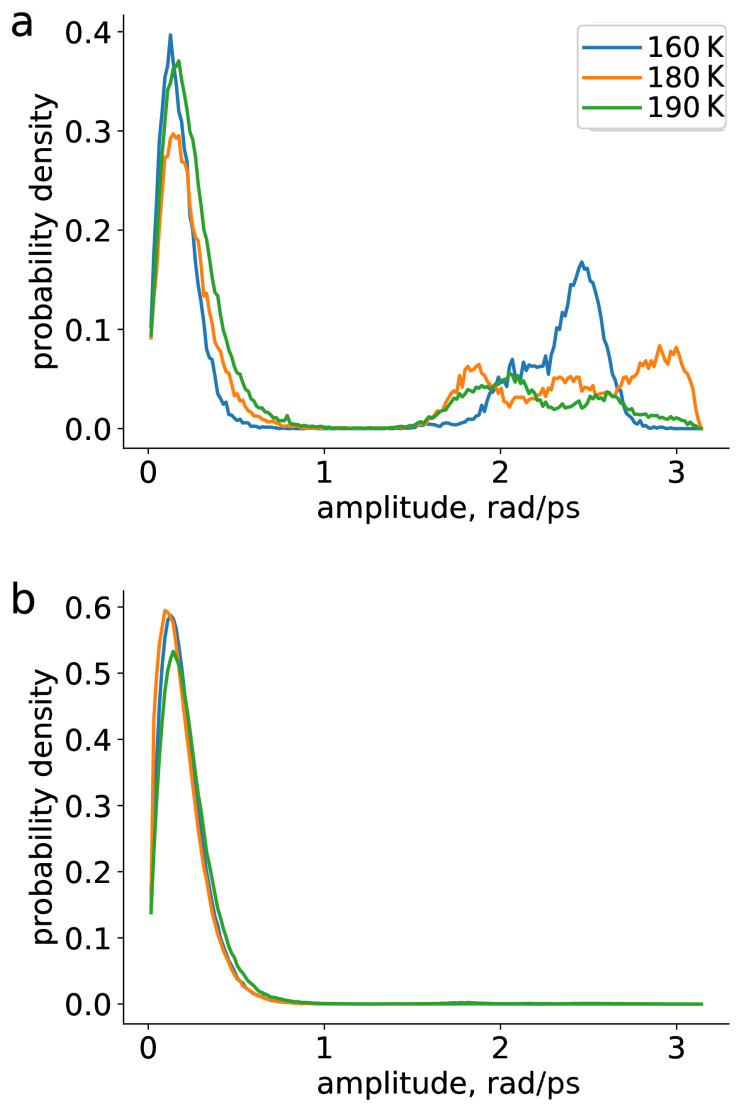
Rotational spectra for mobile (**top**) and immobile (**bottom**) fractions of TEMPO radical with temperature.

Two types of rotational motion could be thus distinguished, corresponding to the peaks of 0.2 and 2.5 rad/ps: librations (small angles) and large amplitude rotational jumps. Geometric properties allowed us to single out two types of TEMPO’s solvation, more analysis is presented below. We looked separately at those trajectories, where the radical was in close contact with O=C fragment (see Figure 2c). In such solvation the motion of the radical was restricted based on its rotational spectra (Figure 4a, <1 rad) as compared to the average (Figure 3). On the other hand, for some trajectories rotation of TEMPO radical on larger angles was possible (Figure 4, >1 rad). For trajectories where the radical was mobile (Figure 4a), both types of rotational motion were observed, whereas for trajectories where the radical was immobilized, only librations were possible.

The number of trajectories corresponding to the mobile and immobile fractions was determined and compared with EPR experiment (Table 1). Two major quantities were determined in EPR vs. temperature: the fraction of mobile radicals exhibiting large-angle rotations *M* (with the fraction of immobile radicals being 1-*M*), and the libration parameter *L* reflecting the intensity of librations ($L∼<a^{2}>t_{c}$, where *a* is the angular amplitude of librations and $t_{c}$ is their characteristic time). The same two parameters can be obtained from the MD data. Table 1 shows excellent agreement in trends of both *M* and *L* parameters vs. temperature obtained by EPR and MD. Namely, monotonic growth and reasonable quantitative agreement was obtained for *M* values, and non-monotonic behavior with local minimum at 180 K was obtained for *L*.

**Figure 5:**
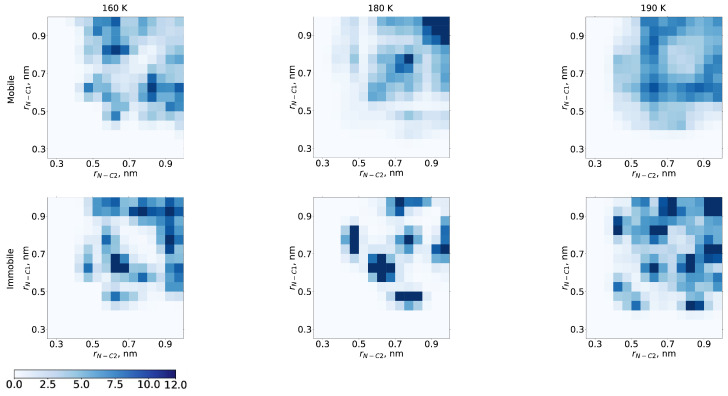
Distribution of distances to chain ends (C1 and C2) of DBP molecules for mobile (**top row**) and immobile fraction (**bottom row**) with temperature 160 K (**left**), 180 K (**middle**), 190 K (**right**). The coordinates correspond to the distances from the first (C1) and second chain ends (C2) of DBP to nitrogen atom of TEMPO (N).

We now proceed to describe differences in solvation pattern for immobile and mobile fractions in more detail. The orientation of the dibutyl phthalate molecules relative to the radical was studied. For this reason, a 2-dimensional distribution of two distances was built—from N to the end of one chain of dibutyl phthalate (C1), and from N to the end of another chain (C2) (Figure 5).

For all temperatures, we observed that the immobile fraction has more defined positions of the DBP chains relative to the TEMPO radical as compared to the mobile fraction. Furthermore, the distribution for immobile fraction at 180 K appeared to be the most structured. Here, mostly one of three possible scenarios was realized: structures with both chains’ termini at ∼0.6 nm, structures with the one chain at ∼0.75 and another at ∼0.45 nm, and vice versa. As was mentioned above, the immobile (‘compressed’) structures also had direct contact between TEMPO and carbonyl oxygen atoms of the DBP molecules. In such a solvation, the radical was almost completely immobilized and could only librate. We believe that the increased contribution of the immobile/compressed fraction lies in the core of the structural anomaly probed by EPR.

Figure S1 shows the two-dimensional RDF, calculated for distances between N atom of the TEMPO and the C atoms of the chains termini or O atoms of DBP. The distribution was calculated for mobile and immobile fractions; however, only the immobile fraction is shown. It turned out that for mobile fraction for any temperature of 160, 180, 190 K, the area of small distances was almost uninhabited. Therefore, only the immobile fraction contributed to the distribution at small distances.

At the same time, on average the mobile fraction had alkyl chains farther away from the radical. This, combined with a smoother distribution of possible chain orientations, allowed for rotational mobility of the radical. Moreover, we found that large-angle TEMPO rotations were coupled with alkyl chain motions. We looked into the MD trajectories during 0–7 ps prior to large-scale rotation of TEMPO and observed that the chain moved farther away from the radical allowing the latter to perform this rotation (see Figure S2, the data were averaged over all rotations). Interestingly, the RDF changed between seven and three picoseconds prior to the rotation, from this we could estimate a characteristic motion time of 4 ps for the butyl chain.

**Figure 6:**
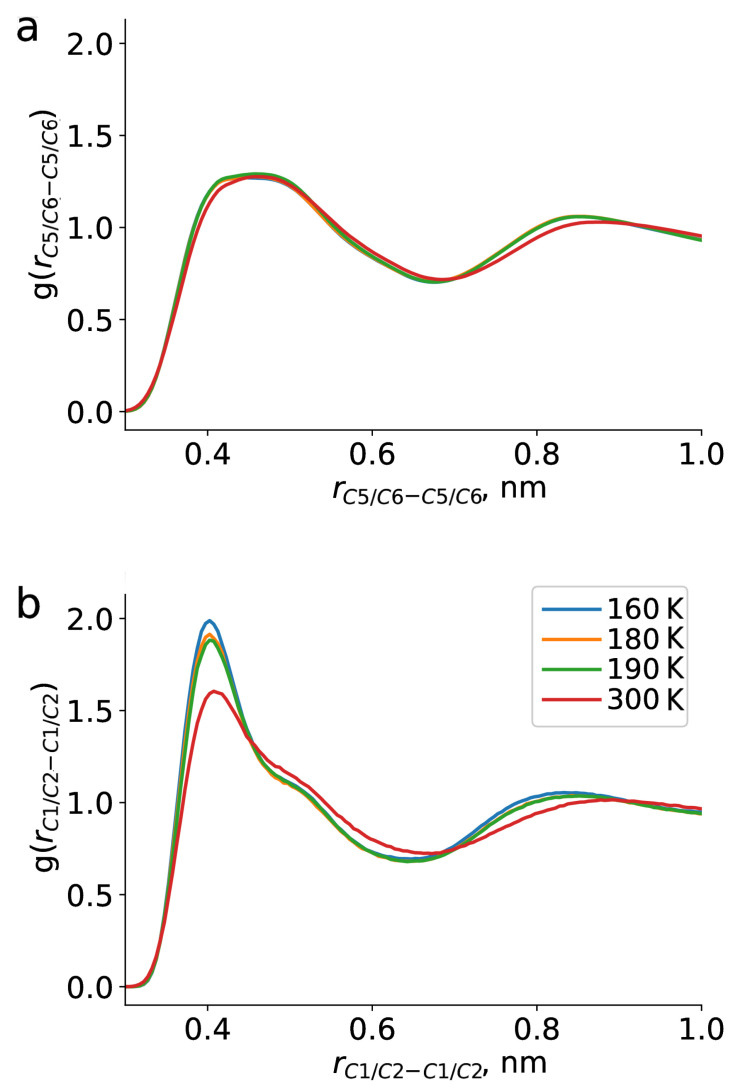
Radial distribution functions for distance between DBP rings (C5 and C6 atoms in Figure 1; (**a**) at different temperatures; (**b**) and DBP chain ends (C1 and C2 atoms in Figure 1).

**Figure 7:**
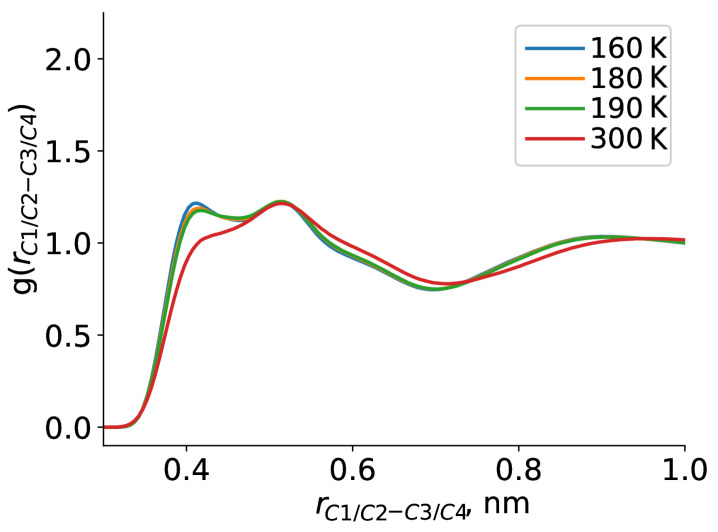
Radial distribution function between C1/C2 and C3/C4 atoms of DBP with temperature.

Apart from TEMPO-DBP interactions, we have also addressed the dynamics of the pure bulk DBP. Figure 6 shows RDFs calculated for this case. First of all, generally similar behaviour was observed compared to TEMPO-DBP results: the peaks became narrower and shifted toward the shorter distances as the temperature was lowered. Next, we investigated temperature changes occurring in the bulk DBP and their relevance to the type of environment sensed by TEMPO radical. Relative positions of the rings did not show temperature dependence (see Figure 6a). First broad peak of the RDF showed presence of some structures with direct contact between the rings, which was of course expected for aromatic systems. However, we did not observe an increase in population of such structures at lower temperature. On the other hand, RDFs between the ends of the alkyl chains of DBP did change with temperature. With temperature decrease the peaks on the RDF become more narrow and intense: occurrence of two chain ends in direct contact grew from 1.6 at 300 K to 2.0 at 160 K relative to the bulk. Interestingly, relative arrangement of the alkyl chains was also found to be temperature dependent, which is shown in RDF between C1/C2 to C3/C4 in Figure 7, only inter-molecular distances were considered. From this RDF we observed an increased population of C1/C2 atoms being in close vicinity of the C3/C4 atoms at lower temperatures. We interpreted this as anti-parallel arrangement of alkyl chains and formation of clusters.

Figure S3 shows the distribution of clusters’ sizes. We considered two types of clusters: when ends of butyl chains (C1/C2) were in close contact or when end of a chain of one molecule (C1/C2) and the (C3/C4) atom of another molecule were in close contact. Butyl chains of DBP were assigned to a cluster when the distance between considered atoms was less than 0.42 nm (based on their Bondi radii), only inter-molecular distances were taken into account. The mean size of the cluster on both occasions depended monotonically on temperature, being the largest at 160 K and then gradually decreased at 180, 190 and 300 K. The largest clusters were formed at 160 K, these were clusters of the second type with an average of 7.5 butyl tails, and they might refer to the average 7.5 or fewer DBP molecules. In summary, we saw a strong structural heterogeneity of the DBP glass. Two types of clusters were formed and corresponded to regions with increased density. The clusters contained only a small proportion of all DBP molecules, the rest were in an unstructured phase. However, despite an extensive sampling we could not observe temperature irregularities in the bulk DBP in contrast to TEMPO-DBP interactions. We would like to note here that the length of every low temperature trajectory was 15 ns, which might be too short to account for a full structural relaxation of DBP glass [5].

## 3. Materials and Methods

Our MD simulations were performed using the GROMACS simulation package [30]. Initial OPLS-AA [31] force field parameters were obtained from LigParGen [32] and then optimized based on BLYP/def2-TZVP [33] calculations by ORCA [34]. Force field parameters for the TEMPO radical were adapted from Sezer et al. [35] MD simulations were performed in a cubic periodic box with a side length of 6 nm at room temperature. Single TEMPO molecule was solvated in 500 DBP molecules. We performed a 400 ns NVT run at room temperature with a 1 fs timestep, prior to this the cell was relaxed and pressure equilibrated (to 1 bar) for 10 ns. We used a CSVR [36] thermostat and Berendsen [37] barostat. Every 5 ns a snapshot was extracted from the room temperature trajectory. The temperature was then annealed separately to 160, 180, and 190 K in the NPT ensemble over 1 ns, followed by a NVT run of 15 ns, where only the last 10 ns were used for the data analysis. In total 81 snapshots were annealed, resulting in 1.215 μs of data sampling for every low temperature. We used MDTraj [38] and VMD [39] for data analysis.

## 4. Conclusions

We performed force-field MD simulations of the TEMPO radical in DBP at 300, 190, 180 and 160 K, respectively. Interplay of complex interactions in the glass resulted in a very heterogeneous solvation pattern of the radical, which was experimentally probed by EPR spectroscopy in a previous work [14]. Here, we observed two distinct solvation patterns of the radical. When the radical was solvated by alkyl chains and had ester groups in its direct vicinity, the rotational motion was constrained. The second solvation type also occurred in the region of alkyl chains: it allowed for not only librations, but also large-angle rotations. For this mobile fraction, we have demonstrated that the rotation of TEMPO was coupled to the motion of butyl chains. Proportion of immobile structures was the largest near the glass transition temperature. Overall, we believe that these effects stand behind the experimentally observed structural anomaly. In addition, a formation of two types of clusters in bulk DBP was observed.

In summary, we would like to emphasize that various radicals’ solvations could occur in glass, leading to completely different regimes of motion observed at the same temperature. Our findings underline the importance of having a clear atomistic picture for interpretation of experimental results in organic glasses near their glass transitions.

## Figures and Tables

**Table 1:** Comparison of parameters of mobility and libration measured in EPR and MD experiments vs. temperature. The error of both experimental and calculated values does not exceed ∼10%.

T,	M${}_{\mathrm{EPR}}$,	L${}_{\mathrm{EPR}}$,	M${}_{\mathrm{MD}}$,	L${}_{\mathrm{MD}}$, $\times 10^{2}$
K	a.u.	a.u.	a.u.	rad${}^{2}$/ps
160	0	2.3	0.06	7.5
180	0.2	2.1	0.17	6.3
190	0.4	3.3	0.33	14.5

## Data Availability

Data can be obtained from the corresponding authors per e-mail.

## Supplementary Materials

The following supporting information can be downloaded at: https://www.mdpi.com/article/10.3390/ijms232314859/s1.

## References

[1] Sillescu H. (1999). Heterogeneity at the glass transition: A review. J. Non-Cryst. Solids.

[2] Wisitsorasak A., Wolynes P.G. (2014). Dynamical Heterogeneity of the Glassy State. J. Phys. Chem. B.

[3] Ediger M.D. (2000). Spatially Heterogeneous Dynamics in Supercooled Liquids. Annu. Rev. Phys. Chem..

[4] Biroli G., Garrahan J.P. (2013). Perspective: The glass transition. J. Chem. Phys..

[5] Brace D.D., Gottke S.D., Cang H., Fayer M.D. (2002). Orientational dynamics of the glass forming liquid, dibutylphthalate: Time domain experiments and comparison to mode coupling theory. J. Chem. Phys..

[6] Wang Y.L., Li B., Sarman S., Mocci F., Lu Z.Y., Yuan J., Laaksonen A., Fayer M.D. (2020). Microstructural and Dynamical Heterogeneities in Ionic Liquids. Chem. Rev..

[7] Russina O., Triolo A., Gontrani L., Caminiti R. (2012). Mesoscopic Structural Heterogeneities in Room-Temperature Ionic Liquids. J. Phys. Chem. Lett..

[8] Ji Y., Shi R., Wang Y., Saielli G. (2013). Effect of the Chain Length on the Structure of Ionic Liquids: From Spatial Heterogeneity to Ionic Liquid Crystals. J. Phys. Chem. B.

[9] Khudozhitkov A.E., Stange P., Bonsa A.M., Overbeck V., Appelhagen A., Stepanov A.G., Kolokolov D.I., Paschek D., Ludwig R. (2018). Dynamical heterogeneities in ionic liquids as revealed from deuteron NMR. Chem. Commun..

[10] Ivanov M.Y., Prikhod’ko S.A., Adonin N.Y., Kirilyuk I.A., Adichtchev S.V., Surovtsev N.V., Dzuba S.A., Fedin M.V. (2018). Structural Anomalies in Ionic Liquids near the Glass Transition Revealed by Pulse EPR. J. Phys. Chem. Lett..

[11] Bakulina O.D., Ivanov M.Y., Prikhod’ko S.A., Pylaeva S., Zaytseva I.V., Surovtsev N.V., Adonin N.Y., Fedin M.V. (2020). Nanocage formation and structural anomalies in imidazolium ionic liquid glasses governed by alkyl chains of cations. Nanoscale.

[12] Ivanov M.Y., Prikhod’ko S.A., Bakulina O.D., Kiryutin A.S., Adonin N.Y., Fedin M.V. (2021). Validation of Structural Grounds for Anomalous Molecular Mobility in Ionic Liquid Glasses. Molecules.

[13] Ivanov M.Y., Poryvaev A.S., Polyukhov D.M., Prikhod’ko S.A., Adonin N.Y., Fedin M.V. (2020). Nanoconfinement effects on structural anomalies in imidazolium ionic liquids. Nanoscale.

[14] Ivanov M.Y., Bakulina O.D., Alimov D.V., Prikhod’ko S.A., Veber S.L., Pylaeva S., Adonin N.Y., Fedin M.V. (2021). Inherent heterogeneities and nanostructural anomalies in organic glasses revealed by EPR. Nanoscale Adv..

[15] Canongia Lopes J.N.A., Pádua A.A.H. (2006). Nanostructural Organization in Ionic Liquids. J. Phys. Chem. B.

[16] Wang Y., Voth G.A. (2005). Unique Spatial Heterogeneity in Ionic Liquids. J. Am. Chem. Soc..

[17] Brehm M., Weber H., Thomas M., Hollóczki O., Kirchner B. (2015). Domain Analysis in Nanostructured Liquids: A Post-Molecular Dynamics Study at the Example of Ionic Liquids. ChemPhysChem.

[18] Gehrke S., von Domaros M., Clark R., Hollóczki O., Brehm M., Welton T., Luzar A., Kirchner B. (2018). Structure and lifetimes in ionic liquids and their mixtures. Faraday Discuss..

[19] Oganesyan V.S. (2018). EPR spectroscopy and molecular dynamics modelling: A combined approach to study liquid crystals. Liq. Cryst..

[20] Hu Z., Margulis C.J. (2006). Heterogeneity in a room-temperature ionic liquid: Persistent local environments and the red-edge effect. Proc. Natl. Acad. Sci. USA.

[21] Huang X., Margulis C.J., Li Y., Berne B.J. (2005). Why Is the Partial Molar Volume of CO_2_ So Small When Dissolved in a Room Temperature Ionic Liquid? Structure and Dynamics of CO_2_ Dissolved in [Bmim+] [PF6-]. J. Am. Chem. Soc..

[22] Margulis C.J., Stern H.A., Berne B.J. (2002). Computer Simulation of a “Green Chemistry” Room-Temperature Ionic Solvent. J. Phys. Chem. B.

[23] Araque J.C., Hettige J.J., Margulis C.J. (2015). Modern Room Temperature Ionic Liquids, a Simple Guide to Understanding Their Structure and How It May Relate to Dynamics. J. Phys. Chem. B.

[24] Amith W.D., Araque J.C., Margulis C.J. (2020). A Pictorial View of Viscosity in Ionic Liquids and the Link to Nanostructural Heterogeneity. J. Phys. Chem. Lett..

[25] Rumble C.A., Kaintz A., Yadav S.K., Conway B., Araque J.C., Baker G.A., Margulis C., Maroncelli M. (2016). Rotational Dynamics in Ionic Liquids from NMR Relaxation Experiments and Simulations: Benzene and 1-Ethyl-3-Methylimidazolium. J. Phys. Chem. B.

[26] Amith W.D., Araque J.C., Margulis C.J. (2021). Relationship between the Relaxation of Ionic Liquid Structural Motifs and That of the Shear Viscosity. J. Phys. Chem. B.

[27] Li Q., Deng X., Liu Y., Cheng Q., Liu C. (2021). Gelation of waxy crude oil system with ethylene-vinyl acetate on solid surface: A molecular dynamics study. J. Mol. Liq..

[28] Cook R.L., King H.E., Herbst C.A., Herschbach D.R. (1994). Pressure and temperature dependent viscosity of two glass forming liquids: Glycerol and dibutyl phthalate. J. Chem. Phys..

[29] Dufour J., Jorat L., Bondeau A., Siblini A., Noyel G. (1994). Shear viscosity and dielectric relaxanon time of dibutyl phthalate down to glass transition temperature. J. Mol. Liq..

[30] Lindahl A., van der Spoel H. (2020). GROMACS 2020 Source Code.

[31] Jorgensen W.L., Maxwell D.S., Tirado-Rives J. (1996). Development and Testing of the OPLS All-Atom Force Field on Conformational Energetics and Properties of Organic Liquids. J. Am. Chem. Soc..

[32] Dodda L.S., Cabeza de Vaca I., Tirado-Rives J., Jorgensen W.L. (2017). LigParGen web server: An automatic OPLS-AA parameter generator for organic ligands. Nucleic Acids Res..

[33] Weigend F., Ahlrichs R. (2005). Balanced basis sets of split valence, triple zeta valence and quadruple zeta valence quality for H to Rn: Design and assessment of accuracy. Phys. Chem. Chem. Phys..

[34] Neese F. (2018). Software update: The ORCA program system, version 4.0. WIREs Comput. Mol. Sci..

[35] Sezer D., Freed J.H., Roux B. (2008). Parametrization, Molecular Dynamics Simulation, and Calculation of Electron Spin Resonance Spectra of a Nitroxide Spin Label on a Polyalanine *α*-Helix. J. Phys. Chem. B.

[36] Bussi G., Donadio D., Parrinello M. (2007). Canonical sampling through velocity rescaling. J. Chem. Phys..

[37] Berendsen H.J.C., Postma J.P.M., van Gunsteren W.F., DiNola A., Haak J.R. (1984). Molecular dynamics with coupling to an external bath. J. Chem. Phys..

[38] McGibbon R.T., Beauchamp K.A., Harrigan M.P., Klein C., Swails J.M., Hernández C.X., Schwantes C.R., Wang L.P., Lane T.J., Pande V.S. (2015). MDTraj: A Modern Open Library for the Analysis of Molecular Dynamics Trajectories. Biophys. J..

[39] Humphrey W., Dalke A., Schulten K. (1996). VMD—Visual Molecular Dynamics. J. Mol. Graph..

