# Supplementary material for: Structural Anomaly in Glasses: Molecular Dynamics Study of Organic Radical in Dibutylphthalate at Different Temperatures
Source: Int J Mol Sci. 2022 Nov 28;23(23):14859. doi: 10.3390/ijms232314859 (PMC9735606; doi:10.3390/ijms232314859)
Supplement: Supplementary file 1 [file ijms-23-14859-s001.zip › ijms-2005045-supplementary.pdf]

Structural Anomaly in Glasses: Molecular Dynamics Study of organic radical in Dibutylphthalate at Different Temperatures

Dmitry V. Alimov,[†] Mikhail Yu. Ivanov,[†] Svetlana Pylaeva,[*,‡] and Matvey V. Fedin[*,†]

†*International Tomography Center, Siberian Branch of the Russian Academy of Sciences, Novosibirsk, 630090, Russia and Novosibirsk State University, Novosibirsk, 630090, Russia*

‡*NMR Spectroscopy group, Bijvoet Center for Biomolecular Research, Utrecht University, Padualaan 8, 3584CH, Utrecht, The Netherlands*

E-mail: s.pylaeva@uu.nl; mfedin@tomo.nsc.ru

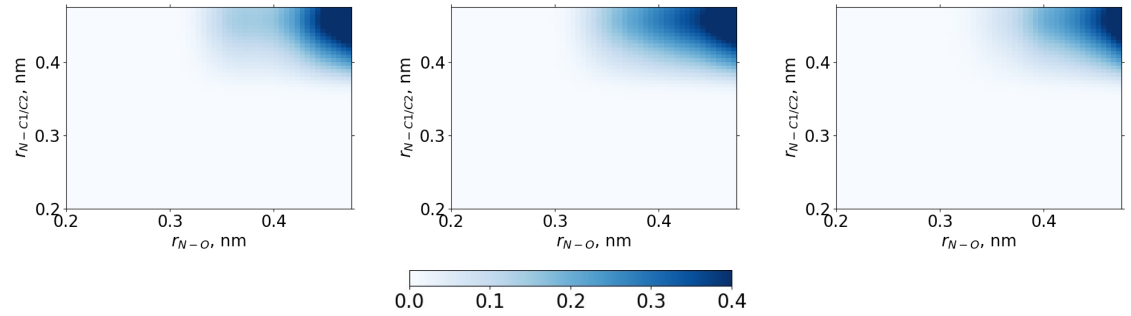

Figure S1: Distribution of distances to chain ends (C1 and C2) and O atoms of DBP molecules for immobile fraction with temperature 160 K(left), 180 K(middle), 190 K(right). The coordinates correspond to the distances from the chain ends (C1/C2) and from the O atoms of DBP to nitrogen atom of TEMPO (N).

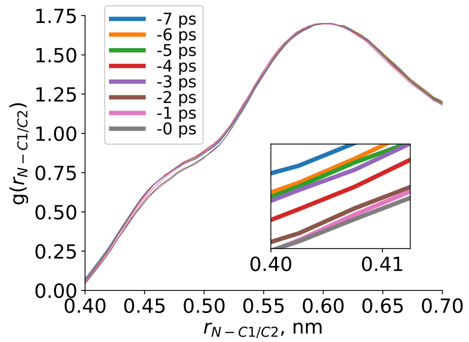

Figure S2: $g(r_{N-C1/C2})$ for a mobile fraction of radicals at different times before a large amplitude rotation of the TEMPO.

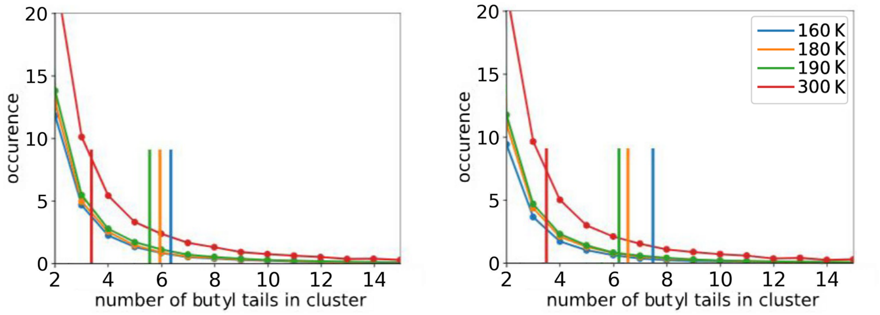

Figure S3: Distribution of DBP cluster sizes. In our study two types of clusters have been considered: the ones formed by the ends of butyl tails (left), and the ones formed by close contacts of C1/C2 atoms of one molecule and C3/C4 atom of another one (right). Atoms are marked in Figure S1. The error of mean value measurement (root-mean-square deviation) does not exceed ∼1%.

Note that in both cases shown in Figure S3 (left and right plots) we have analyzed the behavior of each butyl chain separately, i.e., without verification of whether two chains belong to the same DBP molecule or to the different ones. Therefore, N butyl tails might refer to N or less DBP molecules, since each DBP molecule has 2 butyl tails and either 1 or 2 tails might theoretically become engaged in the cluster. Note, however, that the situation shown on the right most likely refers to the close contacts of different molecules, since the contacts of C1/C2 atoms with C3/C4 atom within a single molecule are sterically unfavored.
